# Sequence and phylogenetic analysis of the mitochondrial genome for the East Asian common octopus, *Octopus sinensis* (Octopodidae: Octopoda)

**DOI:** 10.1080/23802359.2021.1944360

**Published:** 2021-06-28

**Authors:** Fenghui Li, Yuyan Liu, Bo Qin, Li Bian, Jianlong Ge, Qing Chang, Hui Liu, Siqing Chen

**Affiliations:** a Yellow Sea Fisheries Research Institute, Chinese Academy of Fishery Sciences, Laboratory for Marine Fisheries Science and Food Production Processes, Pilot National Laboratory for Marine Science and Technology (Qingdao), Qingdao, China; b National Demonstration Center for Experimental Fisheries Science Education, Shanghai Collaborative Innovation for Aquatic Animal Genetics and Breeding, Shanghai Engineering Research Center of Agriculture, Shanghai Ocean University, 999 Huchenghuan Road, Lingang New District, Shanghai, China; c East China Sea Fisheries Research Institute, Chinese Academy of Fishery Sciences, Shanghai, China

**Keywords:** mtDNA, *Octopus sinensis*, mitochondrion, phylogenetic analysis

## Abstract

The complete mitochondrial genome of the East Asian common octopus (*Octopus sinensis*) was determined and analyzed in this work. The circular mitogenome of *O. sinensis* is 15,737 bp in length with 21.53% GC content, which contains two ribosomal RNA genes (rRNAs), 22 transfer RNA genes (tRNAs), 13 protein-coding genes (PCGs) and a non-coding region (D-loop). The analysis of the SNPs revealed 4 conservative SNPs for COI gene of *O. sinensis* compared with *Octopus vulgaris*. Phylogenetic analysis suggested that *O. sinensis* is closely related to *O. vulgaris*. This sequence data would play an important role in the investigation of phylogenetic relationship and taxonomy of the class Cephalopoda.

The East Asian common octopus (*Octopus sinensis*) is one of the commercially important cephalopods in China. It distributes mainly in the shallow temperate waters of the western North Pacific oceans, particularly in the coastal regions of South Korea, China and Japan (Gleadall 2016). Due to the morphological similarity, it was previously synonymized with the common octopus (*Octopus vulgaris*) that inhabits in Atlantic and Mediterranean and identified as a new species recently based on the molecular features (Amor et al. 2017). The existence of cryptic species and cryptic diversity made the phylogenetic relationship and taxonomy of the class Cephalopoda still unclear. Mitochondrial markers are ubiquitously used to address phylogenetic questions, and have been used to identify some cryptic species complexes (Hebert et al. 2004). Therefore, to better understand the phylogenetic relationship and facilitate the taxonomical study of the cephalopods, we present the complete mitochondrial genome of *O. sinensis*.

An individual sample of *O. sinensis* was captured from the littoral waters of Zhoushan, Zhejiang Province, China (N29°53′36.98″, E122°18′29.01″) in January 2019. Tissue from this specimen was archived in the Yellow Sea Fisheries Research Institute, Chinese Academy of Fishery Sciences (Tissue: #OS-201901). The kidney tissue was collected and stored in liquid nitrogen until DNA extraction. Genomic DNA was isolated using the cetyltrimethylammonium bromide (CTAB) method and sequenced by PacBio sequel platform. The mtDNA sequences were selected by aligning the total sequences to the NCBI database and aligned using Clustal X software. The conflicted nucleotide regions caused by reduced read accuracy of the PacBio sequencing method were detected and corrected by the sequences amplified with 14 premier sets (Table S1), which were sequenced by Illumina HiSeq X platform (Wenger et al. 2019). This genome was annotated by using MITOS (Bernt et al. 2013). The complete mitochondrial genome sequence of *O. sinensis* is 15,737 bp in length with GC content of 25.13% (GenBank accession no. MT712046), containing 13 protein-coding genes (PCGs), 22 transfer RNA (tRNA) genes, two ribosomal RNA (rRNA) genes and one control region (D-loop). Among the 13 protein-coding genes, except ND3, COX2 and ND5 use an incomplete stop codon ‘T’, the rest are encoded by the typical ‘TAA’ or ‘TAG’ stop codons. The length of the control region (D-Loop) is 713 bp. A total of 40 species were used to construct the phylogenetic tree to confirm the phylogenetic position of *O. sinensis*, among which 38 species were cephalopods and *Crassostrea gigas* and *Mizuhopecten yessoensis* were treated as outgroup. The complete mitochondrial genomes of these species were aligned using Clustal X software (Larkin et al. 2007), and maximum likelihood (ML) analysis was conducted using MEGA 7.0 with 1000 bootstrap replicates based on the model of GTR + F + I + G4 (Kumar et al. 2016). The phylogenetic tree showed that the *O. sinensis* is most closely related to the *O. vulgaris* (Figure 1). DNA barcoding using the mitochondrial cytochrome c oxidase subunit I (COI) gene is one of the tools that was used to identify the interspecific and intraspecific variations of the genus *Octopus* (Amor et al., 2017). Based on the comparison of COI genes from nine *O. sinensis* and nine *O. vulgaris*, we identified 4 conservative SNPs. The result presented in this study would play an important role in the investigation of phylogenetic relationship and taxonomy of the class Cephalopoda.

**Figure 1.**
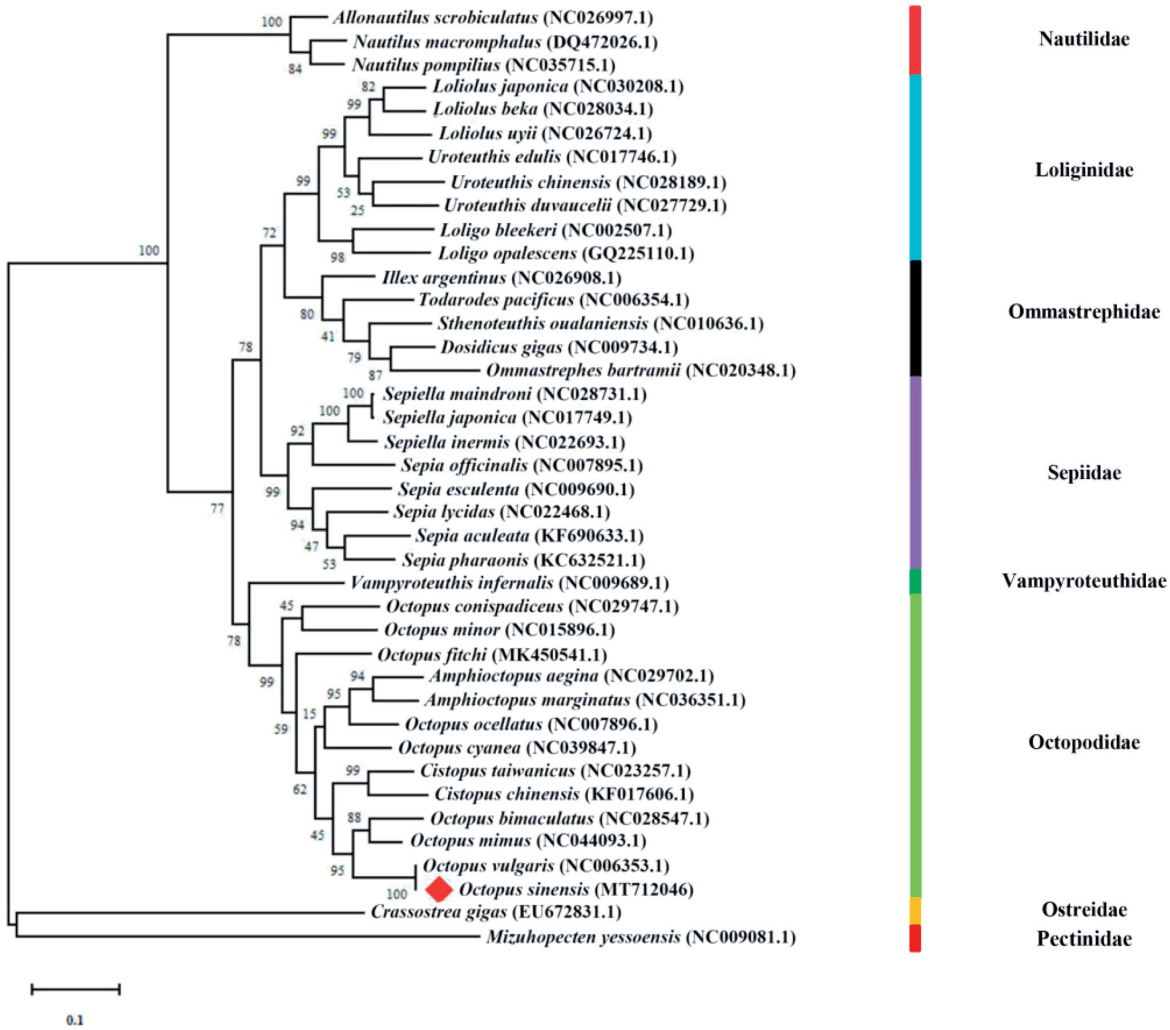
The maximum-likelihood phylogenetic tree based on the complete mitochondrial genomes of 40 species. Bootstrap support is indicated for each branch.

## Data Availability

The genome sequence data that support the findings of this study are openly available in GenBank of NCBI at https://www.ncbi.nlm.nih.gov/nuccore/MT712046.1/ under the accession no. MT712046. The associated BioProject, SRA, and Bio-Sample numbers are PRJNA541812, SRR9265671–SRR9265709, and SAMN11633729, respectively

## References

[CIT0001] Amor MD, Norman MD, Roura A, Leite TS, Gleadall IG, Reid A, Perales-Raya C, Lu CC, Silvery CJ, Vidal EAG, et al. 2017. Morphological assessment of the *Octopus vulgaris* species complex evaluated in light of molecular‐based phylogenetic inferences. Zool Scr. 46(3):275–288.

[CIT0002] Bernt M, Donath A, Jühling F, Externbrink F, Florentz C, Fritzsch G, Pütz J, Middendorf M, Stadler PF. 2013. MITOS: improved de novo metazoan mitochondrial genome annotation. Mol Phylogenet Evol. 69(2):313–319. 22982435 10.1016/j.ympev.2012.08.023

[CIT0003] Gleadall IG. 2016. *Octopus sinensis* d'Orbigny, 1841 (Cephalopoda: Octopodidae): valid species name for the commercially valuable East Asian common octopus. Species Diversity. 21(1):31–42.

[CIT0004] Hebert D, Penton H, Burns JM, Janzen DH, Hallwachs W. 2004. Ten species in one: DNA barcoding reveals cryptic species in the neotropical skipper butterfly *Astraptes fulgerator*. Proc Natl Acad Sci USA. 101(41):14812–14817. 15465915 10.1073/pnas.0406166101 PMC522015

[CIT0005] Kumar S, Stecher G, Tamura K. 2016. MEGA7: molecular evolutionary genetics analysis version 7.0 for bigger datasets. Mol Biol Evol. 33(7):1870–1874. 27004904 10.1093/molbev/msw054 PMC8210823

[CIT0006] Larkin M, Blackshields G, Brown N, Chenna R, McGettigan P, McWilliam H, Valentin F, Wallace I, Wilm A, Lopez R, et al. 2007. Clustal W and Clustal X version 2.0. Bioinformatics. 23(21):2947–2948. 17846036 10.1093/bioinformatics/btm404

[CIT0007] Wenger AM, Peluso P, Rowell WJ, Chang PC, Hall RJ, Concepcion GT, Ebler J, Fungtammasan A, Kolesnikov A, Olson ND, et al. 2019. Accurate circular consensus long-read sequencing improves variant detection and assembly of a human genome. Nat Biotechnol. 37(10):1155–1162. 31406327 10.1038/s41587-019-0217-9 PMC6776680

